# How do physiological networks respond to normobaric hypoxia and isometric exercise?

**DOI:** 10.1113/EP093077

**Published:** 2025-11-20

**Authors:** Danilo Bondi, Cecilia Morandotti, Salvatore Annarumma, Carmen Santangelo, Tiziana Pietrangelo, Stefania Fulle, Vittore Verratti

**Affiliations:** ^1^ Department of Neuroscience, Imaging and Clinical Sciences University ‘G. d'Annunzio’ Chieti‐Pescara Chieti Italy; ^2^ Network Physiology Laboratory, UCL Division of Medicine University College London London UK; ^3^ Department of Biomolecular Sciences University of Urbino Urbino Italy; ^4^ Department of Science University ‘G. d'Annunzio’ Chieti‐Pescara Chieti Italy

**Keywords:** hypoxic stress test, information theory, network comparison, network mapping, network physiology, transfer entropy

## Abstract

The dynamics of physiological systems are impacted by both exercise and hypoxia. Network models can be used to map the interactions between various physiological components in environmental physiology and exercise using the concepts of information theory. This cross‐over study compared three normobaric conditions: control, simulated altitude of 2500 m (fraction of inspired oxygen: $F_{iO_{2}}$ ≈ 15.1%) and 3500 m ($F_{iO_{2}}$ ≈ 13.5%), and rest vs. isometric exercise through the lens of network physiology. The 12 participants (6 M and 6 F; 22.25 ± 2.42 years; 23.01 ± 3.24 kg/m^2^) spent ∼30 min in a tent coupled to an altitude simulator, whose last 3 min consisted of a series of nine unilateral isometric maximal contractions of quadriceps. A metabolic system in breath‐by‐breath mode was used to register cardiorespiratory variables. In‐degree, out‐degree, and transfer entropy (TE) were computed to capture the information flow between variables. A weighted Jaccard Similarity Index was used to assess network similarities. The increase of ${\dot{V}}_{O_{2}}$ in exercise over rest was slightly more prominent during hypoxia (*P *= 0.054, η^2^_p_ = 0.232). Normoxia–hypoxia networks were more similar during resting than exercise. Rest–exercise networks were less similar to each other during simulated altitude of ∼2500 m (*P *= 0.008, η^2^_p_ = 0.353). Neither TE during rest nor during exercise nor the $S_{pO_{2}}$/$F_{iO_{2}}$ ratio significantly predicted the occurrence of symptoms. Unexpectedly, compared to mild‐grade hypoxia, low‐grade hypoxia induced more changes in physiological connectivity, with the majority of the connections converging on putative hidden nodes that we suggest are oxygen delivery‐dependent. Network approaches could offer new developments in exercise and environmental physiology.

## Abstract (highlights)

- **What is the central question of this study?**
  Can network models reveal original findings on how physiological systems are interconnected integrated stressors exposure to hypoxia and exercise?
- **What is the main finding and its importance?**
  The low‐grade hypoxia promoted changes in physiological interconnectedness more than mild grade hypoxia; physical effort triggers physiological variability differentially across hypoxic conditions.

## 1 INTRODUCTION

Humans exposed to hypoxia experience a plethora of physiological changes (West, 2016). The cardiorespiratory system plays a fundamental role in responding to the metabolic demands of the body, thus linking oxygen sensing to emerging adjustments needed to compensate for the lack of oxygen (Zoccal et al., 2024). Addition of another physiological stressor such as physical exercise further affects the dynamics of the physiological systems. Exercise inherently produces a spectrum of effects that are integrated across various physiological systems and subsystems. To deal with multi‐level interconnectedness, network physiology has emerged as a new language for mapping dynamic interactions among dynamically changing entities that give rise to global and emergent states (Ivanov, 2021). Within the broader framework of network physiology, the branch called ‘network physiology of exercise’ focuses on multilevel interactions among physiological systems and multilayer network‐based mechanisms underlying exercise‐related phenomena, thus complementing the continuously growing insights from molecular exercise physiology (Balagué et al., 2022). Molecular exercise physiology usually focuses on non‐dynamical bottom‐up inference, while network physiology of exercise focuses on the nested dynamics of physiological interactions. As outlined by Balagué et al. (2022), the progression of research beyond the molecular level in exercise physiology has been driven by several key factors: the increasing adoption of the mesoscopic approach (Bizzarri et al., 2019), which accounts for co‐emergence in physiological processes and remains largely unaffected by microscopic fluctuations; the methodological advances afforded by the dynamical systems perspective; and the application of properties associated with complex adaptive systems, including both intra‐level and inter‐level synergies.

Using time averaged data from physiological monitoring offers only limited insights, because this approach ignores the complexity and irregularities which are mostly observed in physiological systems (Jiang et al., 2021). The rapidly evolving landscape of wearable devices for the non‐invasive monitoring of biological signals (Guk et al., 2019; Heikenfeld et al., 2018) nowadays offers the possibility of simultaneously acquiring signals from different systems, thereby associating them to define links of synchrony and mutual influence. Therefore, interest in studying functional connectivity of different physiological systems in response to environmental challenges has led to the application of network approaches to understand the complex response to hypoxia (Jiang et al., 2021). Physiological complexity can be quantified via mathematical estimates from different signal–analytical domains; one example is the use of several measures of heart rate variability, whose biomathematical conceptualization can offer physiologically relevant insights (Frasch, 2023).

Some phenomena are triggered by both exercise and hypoxia and can be similarly analysed under the lenses of fluctuating signals analysis. For example, isometric exercise increases diastolic pressure, either by mechanical occlusion or by activation of the metaboreflex; similarly, the pressure response increases under conditions of acute hypoxia via the pathway of muscle sympathetic nerve activity, affecting mainly men compared to women (Jacob et al., 2020). Since exercise and hypoxia per se determine complex and dynamic responses that can be captured by network models, network physiology may capture the integrated response of hypoxia and exercise. The study of fluctuating physiological systems benefits from the analysis of information exchange through network mapping (Morandotti, Rigny et al., 2026). In particular, analyses of cardiorespiratory coordination patterns have emerged as a tool to determine cardiorespiratory function and training‐specific response (Garcia‐Retortillo et al., 2019; Oviedo et al., 2021), and to reveal original exercise‐induced effects that are ineffective in terms of conventionally explored variables (Garcia‐Retortillo et al., 2017). To this extent, transfer entropy has been demonstrated as a useful analytical tool to capture the relationship between physiological time series, for example, for cardiorespiratory information transfer (Faes et al., 2014). Therefore, information theory principles can be applied in environmental physiology studies to map the interaction of multiple physiological components (Jiang et al., 2021).

Within this topic, the type of the hypoxic challenge, that is, either normobaric hypoxia (NH) or hypobaric hypoxia (HH), as well as hypoxic dose, duration, biological variables and symptoms of interest all need to be considered for the determination of the individual physiological response to hypoxia and susceptibility to altitude illnesses. Savourey and colleagues suggested that NH tests, to be preferred over HH test for practical reasons, should last at least 30 min (Savourey et al., 2007). Moreover, those authors indicated determination of possible acute mountain sickness (AMS) development in altitude expeditions could be done following an NH test from algorithms that include also peripheral blood content of oxygen, calculated as [Hb] × $S_{pO_{2}}$ × 1.34 (where Hb is haemoglobin and $S_{pO_{2}}$ is peripheral oxygen saturation), thus relying also on blood sampling, although from mini‐invasive procedures. The hypoxia exercise test is the core of the decision tree as reported by Richalet and colleagues for rationalizing the prescription of acetazolamide through personal physiological response to hypoxia (Richalet et al., 2021). Hypoxia exercise tests for predicting altitude pathologies have been investigated with different protocols which vary depending on level of hypoxia (e.g. fraction of inspired oxygen, $F_{iO_{2}}$, of 0.115, 0.12, 0.131, 0.14), type of activity (e.g. cycling, walking), and predictors (e.g. ventilatory response, cardiac response, cerebral oxygenation) (Georges et al., 2022). In any case, hypoxia exercise tests rely on mostly aerobic efforts. Instead, the incorporation of resistance exercise may yield divergent outcomes, as the demands placed on the cardiorespiratory system are markedly different, by requiring usually abrupt and unstained demands (Luu et al., 2016).

### 1.1 Study aims

With this study, we aimed to investigate the dynamic relationships between the physiological systems in response to isometric exercise and different levels of normobaric hypoxia. Thematic studies on hypoxia are usually aimed at examining the product rather than the process of the acute response. Therefore, the present study aimed to decipher the multi‐ component response across intertwined physiological systems to different hypoxic conditioning. We complemented classical statistical approaches with network physiology approaches to observe whether and how fluctuation of physiological data carries information about hypoxic response in healthy young adults during inactivity and a series of maximal isometric contractions. In addition, we aimed to check whether network‐based metrics were associated to the presence of symptoms that may occur in response to hypoxia.

## 2 METHODS

### 2.1 Ethical approval

Experiments were conducted on humans. All participants provided their written consent after being informed about risks of the study, which was approved by the local ethical committee (‘Comitato Etico delle Province di Chieti e Pescara’, document no. 18, 29/07/2021). The study conformed to the standards set by the *Declaration of Helsinki*, except for registration in a database.

### 2.2 Study design and participants

The study had a cross‐over design across three trials consisting of control, moderate and high simulated altitude, on separate days. Participants were randomly assigned an administration order and remained unaware of such an order until the completion of all sessions. Participants with the following medical conditions were excluded from the study: current diagnosis of ischaemic heart disease, including coronary artery disease and angina pectoris; past acute myocardial infarction; chronic obstructive pulmonary disease; psychiatric disorders such as psychosis, neurosis, schizophrenia, depression, alcoholism, substance abuse and neurodegenerative diseases; neurological disorders; respiratory failure diagnosis; uncontrolled hypertension (diastolic >95 mmHg and/or systolic >180 mmHg); and individuals undergoing anticoagulant therapy. The recruited group consisted of 18 young volunteers (nine females and nine males), who took part in the study during the Physiology course in a medical degree programme. None of the participants had previous experience with normobaric hypoxia, nor were they familiar with this setting before the trials. All participants completed the trials, but data from six participants were excluded *post hoc* due to calibration inaccuracy or problems with data acquisition in at least one of the three recordings. The remaining sample included 12 participants (six males and six females, of age 22.25 ± 2.42 years and BMI 23.01 ± 3.24 kg m^−2^).

### 2.3 Procedures

Participants were tested one at a time using a tent coupled with an altitude generator (Everest Summit II, Hypoxico Inc., New York, USA) as to create the NH condition by reducing the oxygen content into the tent while maintaining constant atmospheric pressure. The hypoxic generator produces the hypoxic environment through a tube inserted into the tent. The experimental design included three conditions with different $F_{iO_{2}}$, spaced ∼7 days apart from one session to another. One of the hypoxic conditions was achieved with $F_{iO_{2}}$ ≈ 15.1%, corresponding to ∼2500 m above sea level (a.s.l.); this condition followed the ‘Hypoxia altitude simulation test’ protocol (Dine & Kreider, 2008), or ‘Hypoxic challenge test’, used to evaluate possible hypoxaemia associated with air travel. The other hypoxic condition was achieved with $F_{iO_{2}}$ ≤ 13.6% (≥3500 m a.s.l.,); this condition followed alternative simulated altitude hypoxia protocols (Saugy et al., 2016), and ensured a high‐altitude condition while allowing participants to be able to stay for the entire duration of the protocol. The normoxic condition was maintained with $F_{iO_{2}}$ ≈ 20.9%. The oxygen percentage inside the tent was regularly monitored by using the integrated oximeter (Handi oxygen monitor, Hypoxico).

**FIGURE 1:**
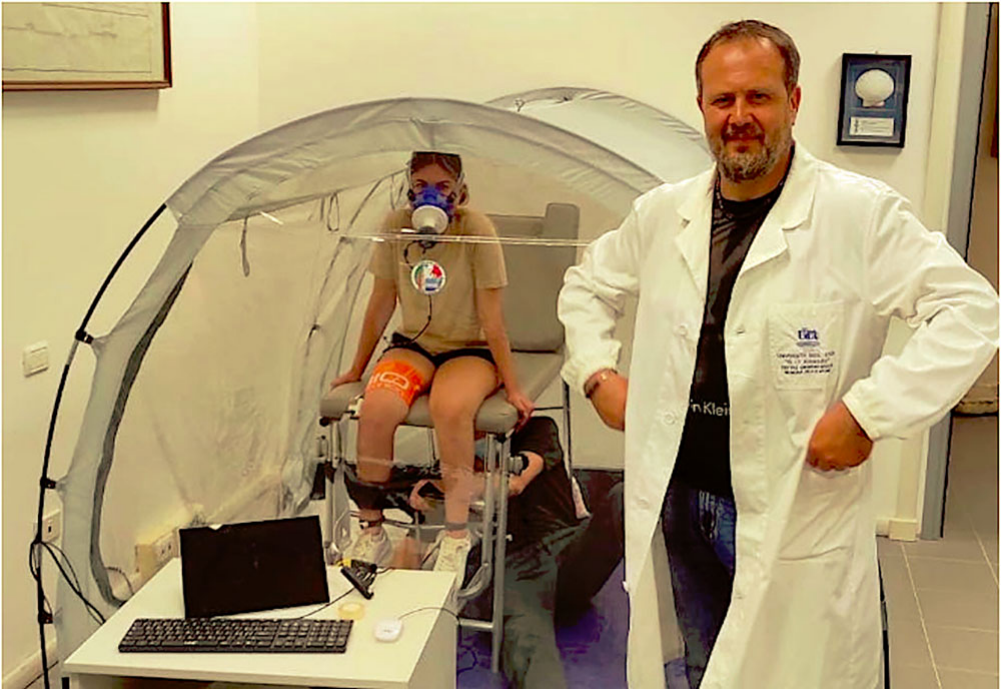
Procedural setting. The hypoxic generator produces the hypoxic environment through a tube inserted into the tent. A medical doctor supervised the experiments. An experimenter entered the tent for 5 min to set and manage the isometric test.

Participants remained in the tent for approximately 30 min. The experimental procedure included a rest phase (10 min), one psychological test (15–20 min), and a final phase of physical testing. The psychological and neuromuscular insights have been published already (Bondi et al., 2025; Gatti et al., 2024). The exercise phase consisted of a series of nine unilateral isometric contractions of the quadriceps at maximum intensity for 5 s, interspersed with 15 s of passive recovery, resulting in a total time of 3 min. During this test, surface electromyography was registered, although the results go beyond the scope of this work and will be reported elsewhere. Before and after each session blood pressure and $S_{pO_{2}}$ were registered. After each session, participants reported subjective symptoms, and they were asked to note any delayed symptoms in the hours and days following. After being equipped with the chest strap and face mask of a portable metabolic system, participants were led into a tent (Figure 1). During tests, $F_{iO_{2}}$ was registered with an oximeter (Hypoxico) at least once every 2 min; the $F_{iO_{2}}$ level was considered to be homogeneous inside the tent. A medical doctor supervised all the experiments.

### 2.4 Portable metabolic system

The device used for this study was the K5 Wearable Metabolic System (COSMED srl, Roma, Italy), in breath‐by‐breath (B×B) mode. The COSMED K5 portable metabolic cart offers precise and reliable measurements, and the B×B mode is preferred over the mixing chamber mode during resting and low intensity exercise (Perez‐Suarez et al., 2018). In B×B mode oxygen consumption and carbon dioxide output are calculated over each breathing cycle; this allows the capturing of rapid changes in respiratory gas exchange and ventilation, but necessitates time delay correction between flow and gas signals, whose errors are magnified at greater breathing frequencies (Winkert et al., 2019). The COSMED K5 is equipped with a gas filter correlation (GFC) sensor for O_2_ (range 0–25%, accuracy ± 0.05% vol), a non‐dispersive infrared (NDIR) sensor for CO_2_ (range 0–10%, accuracy ± 0.05% vol), a bidirectional optoelectronic turbine flow sensor (range 0–300 L/min, resolution 12 mL/min, accuracy ±100 mL/min), an integrated barometer and four integrated sensors for internal and external temperature and humidity. It is coupled via ANT+ transmission with a chest band for monitoring heart rate. The data are managed with OMNIA PC software (COSMED).

Before each session, after a warm‐up period to ensure device temperature was at least 42°C (as required by the instrument manufacturer to ensure proper analysis), all calibration routines were performed according to the manufacturer's instructions. In particular, the flow sensing was calibrated by using the 3 L calibration syringe, CO_2_ sensing was calibrated by using a CO_2_ scrubber, O_2_ sensing was calibrated by using room air and a high precision gas mixture (16.00% O_2_ and 5.00% CO_2_), and time delay was calibrated using the breathing routine determined by the instrument manufacturer.

### 2.5 Data analysis

Relative humidity (RH) and temperature (*T*) were recorded during each assessment. The first 25 min were plotted in order to obtain uniform trends across diverse durations of tests. Then, data of the three sessions were merged across participants by using second order smoothing (10 neighbours) and then merged across conditions by using once again second order smoothing (10 neighbours). Finally, from the latter data a 30‐min plot was created by fitting a straight line with least squares fit method. All data of $S_{pO_{2}}$ and $F_{iO_{2}}$ were plotted by using a linear regression; the $S_{pO_{2}}$ to $F_{iO_{2}}$ ratio was calculated by using percentage values of $S_{pO_{2}}$ and fractional values of $F_{iO_{2}}$.

COSMED K5 data were checked and the $F_{iO_{2}}$ was manually adjusted based on data from an oximeter. This procedure was conducted in agreement with the company, since the calibration procedure cannot be performed in a hypoxic environment.

Individual series of respiratory frequency (RF), tidal volume (*V*_T_), heart rate (HR), oxygen consumption (${\dot{V}}_{O_{2}}$), and end‐tidal pressure of O_2_ ($P_{\mathrm{ET}O_{2}}$) and CO_2_ ($P_{\mathrm{ETC}O_{2}}$) were filtered by removing outliers, fitting and interpolating. Time‐series plots were created by adapting the procedure to each series of data.

### 2.6 Network analysis of physiological time series

#### 2.6.1 Physiological network mapping

Network mapping was used to represent the flow of information within the cardiorespiratory system through network diagrams, where nodes represent physiological variables and edges represent transfer entropy (TE) as described (Morandotti, Wikner et al., 2025). For each physiological variable in each experimental condition, a 5‐min‐long time series was extracted from raw data. Considering that COMSED K5 was set in a breath‐by‐breath model, the sampling frequency of physiological data were related to the breathing frequency (e.g. for a 5‐min series with a breathing frequency of 20 bpm, 100 data points were available). An open‐source MATLAB (MathWorks, Natick, MA, USA) function from PhysioNet (https://www.physionet.org/conTEt/tewp/1.0.0/) was used to calculate TE between all pairs of parallel time series of the following physiological variables; HR, RF, ${\dot{V}}_{E}$, *V*_T_, ${\dot{V}}_{O_{2}}$, $P_{\mathrm{ET}O_{2}}$ and $P_{\mathrm{ETC}O_{2}}$ (Lee et al., 2012; Morandotti, Rigny et al., 2026). A previous report demonstrated that the ideal lag time for TE calculations between cardiorespiratory variables is approximately 15 s which is equivalent of five respiratory cycles (Morandotti, Wikner et al., 2025). In this study, data sampling was event‐based (i.e. each event is a respiratory cycle) and thus a lag of one to five respiratory cycles was used initially to calculate the TEs. To find the optimal time lag, TEs were calculated using lags ranging from one to five respiratory cycles and then graphed; a plateau was observed after a lag of two respiratory cycles, meaning there is no significant change in TE values when using a lag of two or higher (data not shown). Therefore, a lag of five was used for all TE calculations used in network mapping. Although using variable lags would have provided a more comprehensive analysis, a fixed lag was chosen to make physiological interpretation easier. Previous research has found that the memory length of the cardiorespiratory time series ranges from 5 to 25 s (Shirazi et al., 2013). This refers to the temporal dependence within a series of physiological measurements, indicating the extent to which past events influence future ones (Satti et al., 2019). Therefore, it is reasonable to assess how the past of one cardiorespiratory time series affects the future of another within this 5–25 s range. In this study, because the sampling frequency during physiological signal recording was the respiratory cycle, a lag of five respiratory cycles was used. This corresponded to approximately 15 s, which falls within the 5–25 s range for all participants’ time series.

The Monte Carlo method was then used to identify any TE values which were due to randomness and did not represent genuine information transfer between physiological variables (Lee et al., 2012; Morandotti, Rigny et al., 2026). To do so, after having calculated TE from variable *A* to variable *B* (TE(*A*→*B*)), the data points of variable *A*’s time series were shuffled at random for 100 times, resulting in 100 different shuffled (*A*) time series. TE was calculated between each shuffled (*A*) time series and the unshuffled (*B*) time series; a probability distribution of shuffled TEs was obtained. The original TE value from *A* to *B* is considered significant if it is greater than the 95th percentile of the distribution obtained from shuffled (random) TEs. If a TE value is less than the 95th percentile it was considered non‐significant and replaced with 0.

After removing non‐significant TE values, the median TE was calculated for each physiological variable and compiled into a 7 by 7 directional adjacency matrix. This matrix was used to generate a directional network map, where nodes represent physiological variables, and edge thickness is directly proportional to TE values. This was repeated for each experimental condition, resulting in six network maps.

Using a MATLAB function, the matrices were then used to calculate indegree (ID) and outdegree (OD) of each node for all experimental conditions. ID and OD are centrality measures which represent the information input into a node and information output from a node, respectively. In other words, they measure the influence of the network on a node (ID) and the influence of a node on the network (OD). Network diagrams were then plotted in MATLAB with node size directly proportional to either ID or OD values.

#### 2.6.2 Comparison between the networks

Following the classification of Tantardini and colleagues about methods for comparing networks, based on network type (directed vs. undirected, weighted vs. unweighted) and known/unknown node‐correspondence (Tantardini et al., 2019), the Weighted Jaccard Similarity Index (WJSI) was used to evaluate network similarities. Considering *A* and *B* to be two networks constituted by the same nodes and a variable number of edges (expressed as 0 or 1 in adjacency matrices), the JSI herein represents the proportion of shared edges out of the total edges, defined by the formula:

$$\mathrm{JSI}=\;\;\left(\left|A\cap B\right|\right)/\left(\left|A\cup B\right|\right)$$

Instead, WJSI takes into account the weight of edges and is calculated as the sum of minimal divided by the sum of maximal pairwise coefficients, defined by the formula:

$$\mathrm{WJSI}=\sum_{i=1}^{N}\min\left(A_{i},B_{i}\right)/\sum_{i=1}^{N}\max\left(A_{i},B_{i}\right)$$

Both JSI and WJSI ranged from 0 to 1, with 1 indicating perfect similarity (i.e. graph isomorphism).

### 2.7 Statistical analysis

Symptoms and headache occurrence in the three experimental conditions were compared by taking into account the percentage obtained during Control (CTR) and then carrying out a χ^2^ test for comparing symptoms occurrence (yes–no) between simulated altitude of 2500 m (HY1) and 3500 m (HY2) with respect to control, separately. Occurrence of headache during the three experimental conditions was compared through McNemar's contingency test for paired samples. After assumption checks, a series of RM‐ANOVAs were carried out for comparing: (1) WJSI revealing rest–exercise network similarities between the three experimental conditions, (2) WJSI revealing CTR–HY1, CTR–HY2 and HY1–HY2 network similarities during resting phase, and (3) WJSI revealing CTR–HY1, CTR–HY2 and HY1–HY2 network similarities during exercise phase. A Greenhouse–Geisser correction was eventually applied after Mauchly's test for sphericity. After assumption checks, a series of binomial regressions was carried out to test whether total TE during rest and exercise in each experimental condition was associated to the presence of symptoms, separately for the three conditions; normality and VIF values were evaluated as an assumption check; the Akaike information criterion (AIC) and Bayesian information criterion (BIC) were calculated to evaluate fitness of the data with the regression model, *P* and *R* ^2^ for the significance and effect size. Any *P*‐value falling below the 0.05 threshold, that is, the probability of the results occurring by chance was less than 5%, was reported to be statistically significant in the ‘Results’ section.

## 3 RESULTS

### 3.1 Grouped data

**FIGURE 2:**
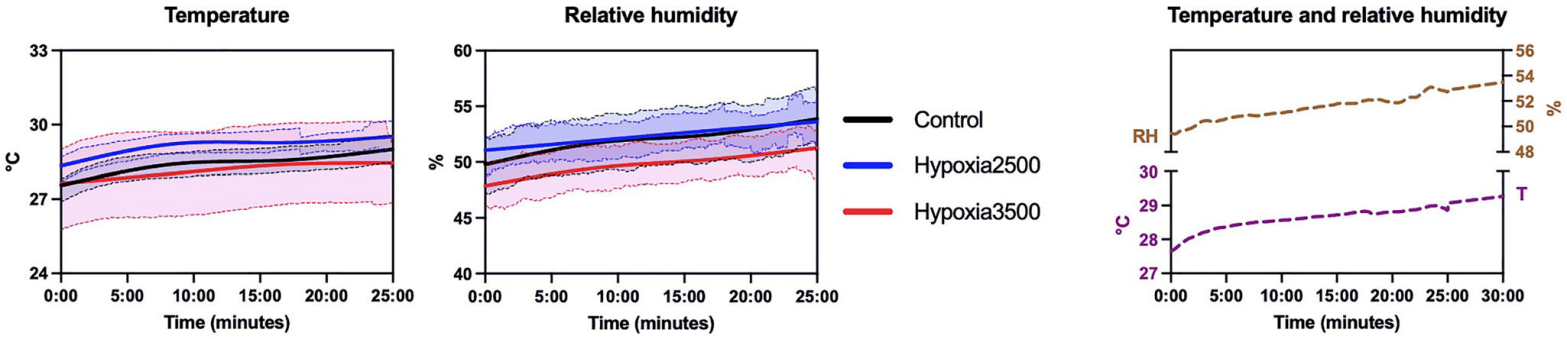
Temperature and relative humidity during the sessions. The first two panel show the observed trends by using a restricted cubic spline method and plotting SEM area around the fitting line. The third panel adds to the first 25 min the predicted last 5 min by using twice a 2nd order smoothing (10 neighbours) and then plotting a straight line by fitting least squares.

RH and *T* trends were similar across conditions and increased during sessions. Figure 2 provides a reference for similar studies; in particular, by using a similar hypoxic tent with one participant at rest, one can expect to find an almost linear rise in *T* and RH with an increase of ∼4% and of ∼1.5°C, respectively, after 30 min.

**FIGURE 3:**
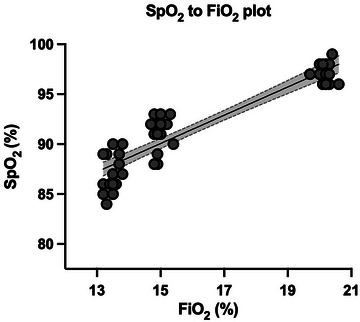
$S_{pO_{2}}$ reduction as triggered by lowering $F_{iO_{2}}$. The graph shows a linear regression line with 95% CI area and all individual data. To obtain more robustness, all data from the initial sample of 18 participants were used.

The $S_{pO_{2}}$ to $F_{iO_{2}}$ ratio (SF) was calculated for all 18 participants. SF increased from control (481 ± 6.40) to simulated 2500 m (609 ± 11.8) and simulated 3500 m (645 ± 13.6), in all participants (*P *< 0.001, η^2^_p_ = 0.987). As shown in Figure 3, the trend of SF followed a linear regression of the equation $S_{pO_{2}}$ = 1.416 × $F_{iO_{2}}$ + 68.83 (*r* = 0.852; *Sy*.*x* = 1.731).

**FIGURE 4:**
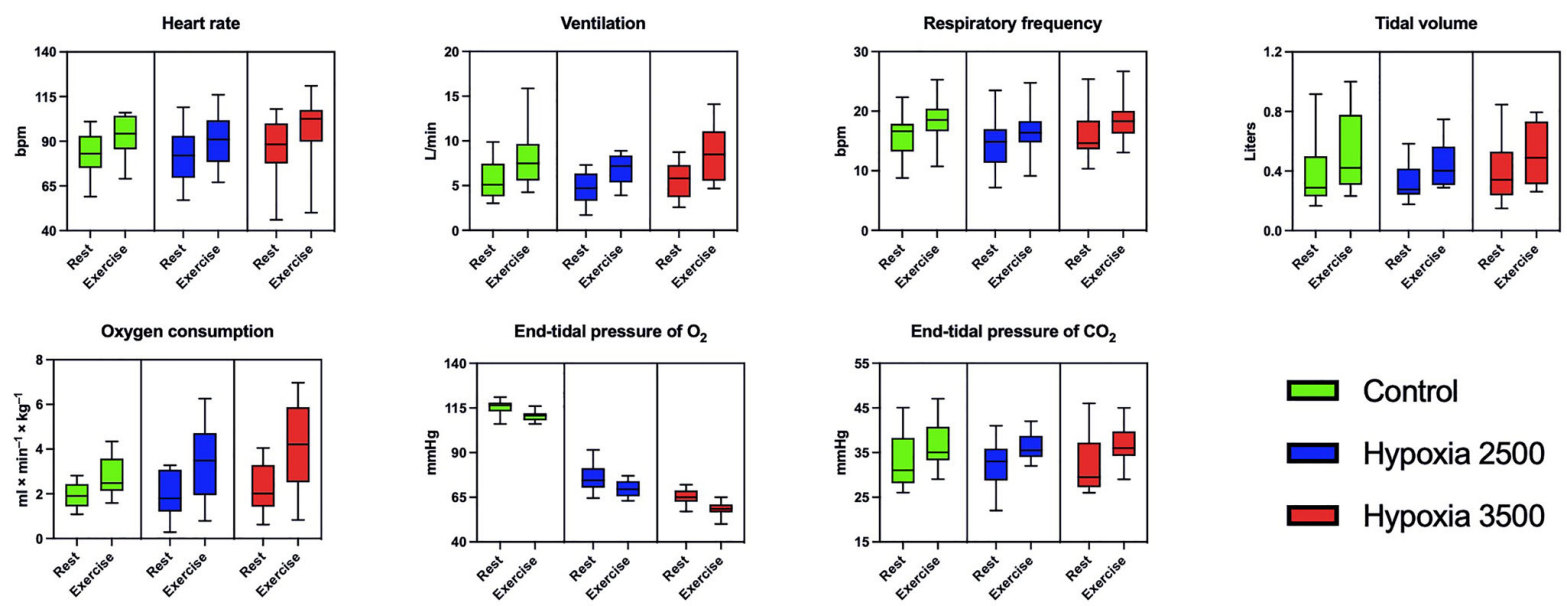
Variables were registered from COSMED K5 and shown as box plots with median, interquartile range and min‐to‐max whiskers, split by rest versus exercise, and coloured by hypoxic conditions. Hypoxia 2500 and 3500 refer the simulated altitudes of 2500 and 3500 m, respectively.

As shown in Figure 4 and Table 1, heart rate (HR) and ventilation (${\dot{V}}_{E}$) did not significantly increase in hypoxia, due to high heterogeneity of responses across participants. The same results were obtained for oxygen consumption (${\dot{V}}_{O_{2}}$), although values tended to increase in hypoxia to a greater extent than HR and ${\dot{V}}_{E}$. There was a clear reduction of end‐tidal pressure of O_2_ ($P_{\mathrm{ET}O_{2}}$) in hypoxia, while end‐tidal pressure of CO_2_ ($P_{\mathrm{ETC}O_{2}}$) did not change. During exercise, ${\dot{V}}_{E}$ increased due to an increase in both respiratory frequency (RF) and tidal volume (*V*_T_), as well as HR and ${\dot{V}}_{O_{2}}$; $P_{\mathrm{ET}O_{2}}$ decreased while $P_{\mathrm{ETC}O_{2}}$ increased during exercise. With regard to factor interaction, the only noteworthy change, although not significant, concerned ${\dot{V}}_{O_{2}}$, whose increase in exercise over rest was slightly more prominent during hypoxia.

**TABLE 1:** Statistical results of grouped data from COSMED K5.

	Hypoxia		Exercise		Interaction	
	*P*	η^2^_p_	*P*	η^2^_p_	*P*	η^2^_p_
HR	0.172	0.148	<0.001	0.880	0.841	0.016
${\dot{V}}_{E}$	0.129	0.170	<0.001	0.913	0.461	0.068
RF	0.240	0.124	<0.001	0.790	0.726	0.029
*V*_T_	0.266	0.113	<0.001	0.862	0.423	0.075
${\dot{V}}_{O_{2}}$	0.105	0.185	<0.001	0.847	0.054	0.232
$P_{\mathrm{ET}O_{2}}$	<0.001	0.985	0.001	0.659	0.381	0.084
$P_{\mathrm{ETC}O_{2}}$	0.941	0.005	0.004	0.540	0.965	0.003

η^2^_p_, partial eta squared, used as effect size; HR, heart rate; ${\dot{V}}_{E}$, ventilation; RF, respiratory frequency; *V*_T_, tidal volume; ${\dot{V}}_{O_{2}}$, Oxygen consumption; $P_{\mathrm{ET}O_{2}}$ and $P_{\mathrm{ETC}O_{2}}$, end‐tidal pressure of O_2_ and CO_2_.

### 3.2 Individual data

**FIGURE 5:**
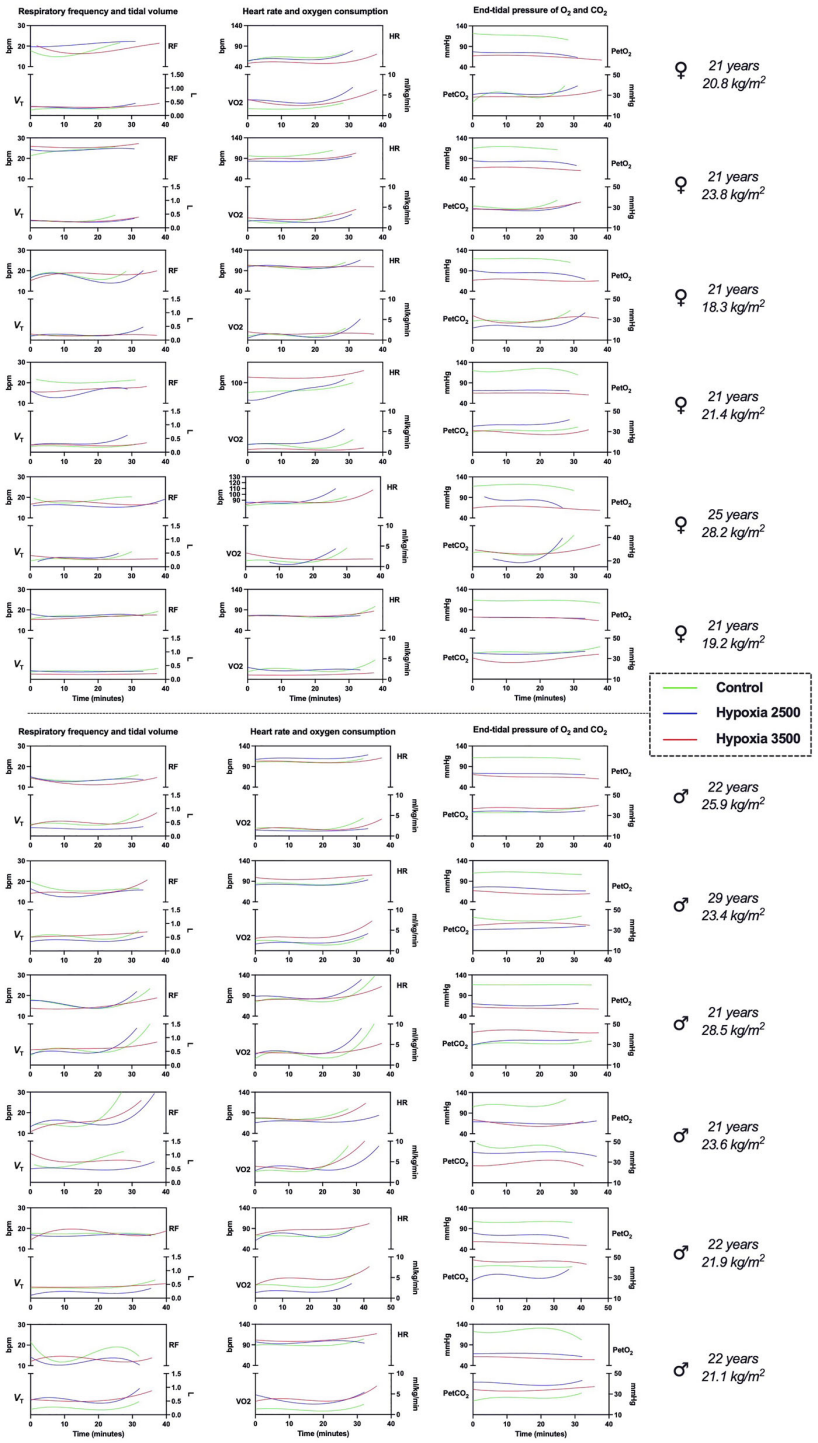
Individual time series plots of data from COSMED K5. Usually, a cubic polynomial method was used; alternatively, fourth order polynomial or LOWESS methods were applied.

The most used method to create a time series plot was the third order polynomial curve; other methods used were second order smoothing (20 neighbours), fourth order polynomial, and locally weighted scatterplot smoothing (LOWESS). The responses to hypoxia, as well as the bodily changes during exercise, were greatly heterogeneous across participants, as shown in Figure 5.

### 3.3 Physiological networks

**FIGURE 6:**
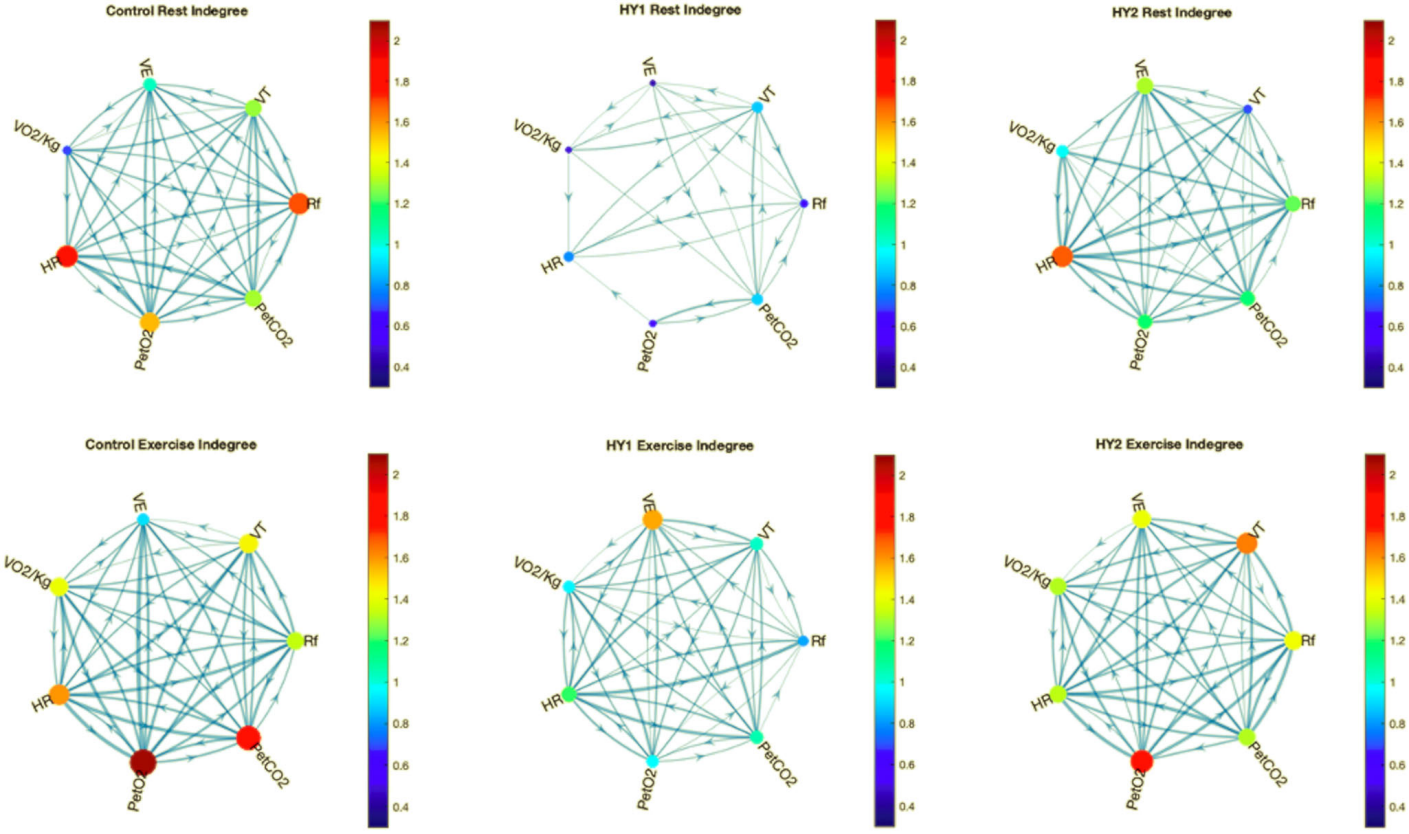
Physiological network diagrams, where node size is directly proportional to ID values and edge thickness is directly proportional to TE values.

The results of TE‐based network mapping showed that in Control–Rest conditions, there was a significant bidirectional flow of information between almost all pairs of nodes (Figure 6). The highest TE values at rest were TE($P_{\mathrm{ET}O_{2}}$→HR) and TE($P_{\mathrm{ETC}O_{2}}$→HR). Significant transfer of information was also seen between $P_{\mathrm{ETC}O_{2}}$→RF, $P_{\mathrm{ETC}O_{2}}$→*V*_T_, *V*_T_→RF, ${\dot{V}}_{E}$→$P_{\mathrm{ET}O_{2}}$, and *V*_T_→$P_{\mathrm{ET}O_{2}}$ (descending order). This is reflected in centrality measures as HR, RF and $P_{\mathrm{ET}O_{2}}$ have the highest indegree (ID) value, whilst ${\dot{V}}_{E}$, $P_{\mathrm{ETC}O_{2}}$ and *V*_T_ have the highest outdegree (OD) values (descending order) (Figure 6 and Supplementary Figure S1).

With the addition of exercise as an intervention (Control–Exercise), changes in transfer of information can be observed in the network. The most significant transfer of information occurred between the nodes of ${\dot{V}}_{E}$→$P_{\mathrm{ET}O_{2}}$, HR→$P_{\mathrm{ET}O_{2}}$, and HR→$P_{\mathrm{ETC}O_{2}}$. The node receiving the most information (ID) in the network was $P_{\mathrm{ET}O_{2}}$, followed by $P_{\mathrm{ETC}O_{2}}$ and HR in Control–Exercise condition. The nodes with the highest information output were HR, RF and ${\dot{V}}_{E}$. This differs from Control–Rest conditions where the highest ID was that of HR due to a higher amount of information directed from other nodes to HR, rather than from HR to other nodes as seen in Control–Exercise.

Further changes are observed in network maps when hypoxia equivalent to 2500 m of altitude (HY1) was introduced. During the HY1–Rest phase, there was a significant reduction in information transfer between all nodes within the network. Although TE values were much lower than those in control conditions, the highest TE values were those between $P_{\mathrm{ETC}O_{2}}$→$P_{\mathrm{ET}O_{2}}$, *V*_T_→HR, *V*_T_→RF, *V*_T_→$P_{\mathrm{ETC}O_{2}}$, HR→*V*_T_, and ${\dot{V}}_{E}$→*V*_T_. This is reflected in centrality measures which showed that *V*_T_ is the node with both the highest input and highest output of information in the network; similarly, $P_{\mathrm{ETC}O_{2}}$ also exhibited relatively high values for both ID and OD.

With the intervention of exercise in HY1 conditions (HY1–Exercise), there is an increase in information transfer between the nodes compared to HY1–Rest, but a slight decrease compared to Control–Exercise. The highest transfer of information occurs between the nodes of HR→RF, HR→$P_{\mathrm{ETC}O_{2}}$, $P_{\mathrm{ETC}O_{2}}$→${\dot{V}}_{E}$, and $P_{\mathrm{ET}O_{2}}$→${\dot{V}}_{O_{2}}$. Again, this is reflected in centrality measures as the nodes which receive the highest amount of information are ${\dot{V}}_{E}$ and HR, whilst those that have the highest output of information are HR and $P_{\mathrm{ETC}O_{2}}$. This is similar to Control–Exercise as HR is the node with the highest OD with high transfer of information from HR to $P_{\mathrm{ETC}O_{2}}$. A difference can be observed between HY1–Exercise and Control–Exercise in the bidirectional flow of information between $P_{\mathrm{ET}O_{2}}$ and ${\dot{V}}_{E}$; in Control–Exercise TE(${\dot{V}}_{E}$→$P_{\mathrm{ET}O_{2}}$) is higher, whilst in HY1–Exercise TE($P_{\mathrm{ET}O_{2}}$→${\dot{V}}_{E}$) is higher.

By increasing the level of hypoxia to an equivalence of 3500 m of altitude (HY2), further changes can be observed in the network's information transfer. During the HY2–Rest phase, it can be observed that the highest TE values are those of RF→$P_{\mathrm{ETC}O_{2}}$ and those of the bidirectional information transfer between HR⇌RF. The nodes receiving the highest amount of information are HR and ${\dot{V}}_{E}$; the nodes with the highest output of information are HR and RF. With the addition of exercise in HY2 conditions, an increase of information transfer can be observed when compared to HY2–Rest and HY1–Exercise. The most prominent flows of information are between $P_{\mathrm{ETC}O_{2}}$→HR, HR→$P_{\mathrm{ET}O_{2}}$, RF→*V*_T_, as well as bidirectionally between $P_{\mathrm{ET}O_{2}}$ and RF. This results in $P_{\mathrm{ET}O_{2}}$, *V*_T_ and RF receiving the highest amount of information, whilst the nodes with the highest output of information are HR, RF and $P_{\mathrm{ETC}O_{2}}$.

The orders of ID and OD values in all experimental conditions are described in Table 2 (ID) and Table 3 (OD); these show how the nodes receiving and releasing the most information changed with different interventions.

**TABLE 2:** Order of ID values of physiological variables for all experimental conditions, in descending order.

Indegree					
Control–Rest	HY1–Rest	HY2–Rest	Control–Exercise	HY1–Exercise	HY2–Exercise
HR	*V*_T_	HR	$P_{\mathrm{ET}O_{2}}$	${\dot{V}}_{E}$	$P_{\mathrm{ET}O_{2}}$
RF	HR	${\dot{V}}_{E}$	$P_{\mathrm{ETC}O_{2}}$	HR	*V*_T_
$P_{\mathrm{ET}O_{2}}$	$P_{\mathrm{ETC}O_{2}}$	RF	HR	${\dot{V}}_{O_{2}}$	RF
*V*_T_	RF	$P_{\mathrm{ET}O_{2}}$	*V*_T_	*V*_T_	$P_{\mathrm{ETC}O_{2}}$
$P_{\mathrm{ETC}O_{2}}$	$P_{\mathrm{ET}O_{2}}$	$P_{\mathrm{ETC}O_{2}}$	${\dot{V}}_{O_{2}}$	$P_{\mathrm{ETC}O_{2}}$	${\dot{V}}_{E}$
${\dot{V}}_{E}$	${\dot{V}}_{E}$	${\dot{V}}_{O_{2}}$	RF	$P_{\mathrm{ET}O_{2}}$	HR
${\dot{V}}_{O_{2}}$	${\dot{V}}_{O_{2}}$	*V*_T_	${\dot{V}}_{E}$	RF	${\dot{V}}_{O_{2}}$

**TABLE 3:** Order of OD values of physiological variables for all experimental conditions, in descending order.

Outdegree					
Control–Rest	HY1–Rest	HY2–Rest	Control–Exercise	HY1–Exercise	HY2–Exercise
${\dot{V}}_{E}$	*V*_T_	HR	HR	HR	HR
$P_{\mathrm{ETC}O_{2}}$	RF	RF	*V*_T_	$P_{\mathrm{ETC}O_{2}}$	$P_{\mathrm{ETC}O_{2}}$
HR	$P_{\mathrm{ETC}O_{2}}$	$P_{\mathrm{ET}O_{2}}$	RF	*V*_T_	RF
*V*_T_	$P_{\mathrm{ET}O_{2}}$	$P_{\mathrm{ETC}O_{2}}$	${\dot{V}}_{E}$	${\dot{V}}_{E}$	${\dot{V}}_{O_{2}}$
$P_{\mathrm{ET}O_{2}}$	${\dot{V}}_{E}$	${\dot{V}}_{E}$	${\dot{V}}_{O_{2}}$	${\dot{V}}_{O_{2}}$	${\dot{V}}_{E}$
${\dot{V}}_{O_{2}}$	HR	*V*_T_	$P_{\mathrm{ET}O_{2}}$	$P_{\mathrm{ET}O_{2}}$	*V*_T_
RF	${\dot{V}}_{O_{2}}$	${\dot{V}}_{O_{2}}$	$P_{\mathrm{ETC}O_{2}}$	RF	$P_{\mathrm{ET}O_{2}}$

**FIGURE 7:**
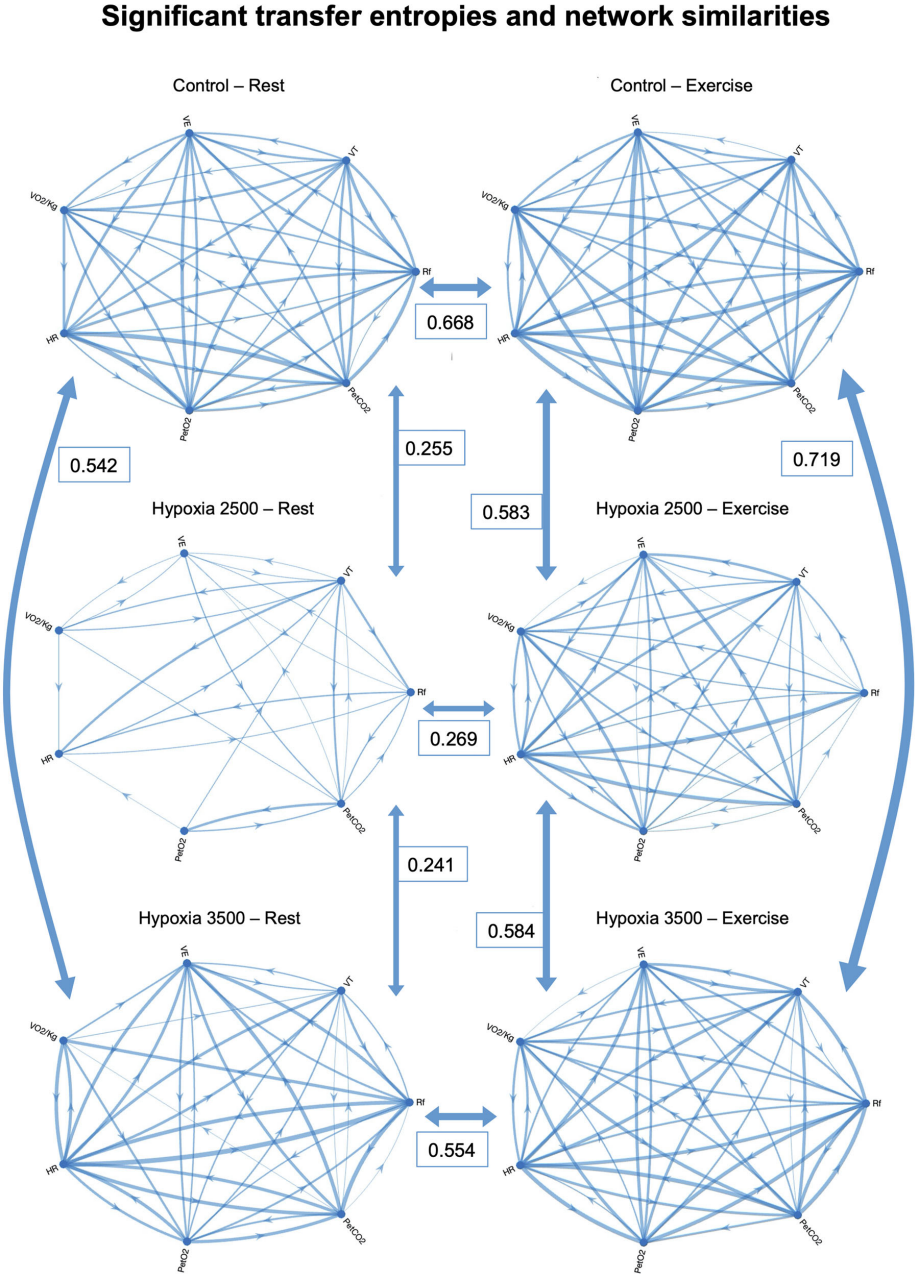
Network similarities as computed with the Weighted Jaccard Similarity Index.

As shown in Figure 7, the most prominent mismatch between rest and exercise network maps (lowest WJSI) was found during simulated altitude of ∼2500 m. The more overlapping the networks shown are, the greater their similarity index, which is calculated for each pair. Across conditions, both at rest and during exercise networks were more similar (greater WJSI) when comparing rest to simulated altitude of ∼3500 m than when comparing the two hypoxic conditions.

As shown in Table 4, the network's total transfer entropy was more associated across conditions during the resting phase than during the exercise phase.

**TABLE 4:** Correlation matrix of transfer entropy in different testing conditions and phases.

		CTR–Rest	CTR–Ex	HY1–Rest	HY1–Ex	HY2–Rest	HY2–Ex
CTR–Rest	Pearson's *r*	—					
	Kendall's τ_B_	—					
CTR–Ex	Pearson's *r*	0.557	—				
	Kendall's τ_B_	0.333	—				
HY1–Rest	Pearson's *r*	0.621*	0.543	—			
	Kendall's τ_B_	0.485*	0.424	—			
HY1–Ex	Pearson's *r*	0.224	0.298	0.294	—		
	Kendall's τ_B_	0.061	0.182	0.273	—		
HY2–Rest	Pearson's *r*	0.680*	0.514	0.687*	0.161	—	
	Kendall's τ_B_	0.515*	0.455*	0.545*	0.061	—	
HY2–Ex	Pearson's *r*	0.385	0.417	0.112	0.403	0.355	—
	Kendall's τ_B_	0.152	−0.030	0.121	0.364	0.212	—

* *P *< 0.05. CTR, control condition; HY1, hypoxia at simulated altitude of ∼2500 m; HY2, hypoxia at simulated altitude of 3500 m; Ex, exercise.

As shown in Table 5, symptoms were present more in HY2 than Control (*P *= 0.014, χ^2^_1d.f._ = 6). Headache was present in few participants in all the conditions. In the same table it is shown how rest–exercise network similarities between the three experimental conditions revealed lower values of WJSI during simulated altitude of ∼2500 m (*P *= 0.008, η^2^_p_ = 0.353). Along the same lines, TE networks of control and hypoxia at ∼3500 m were more similar to each other than to hypoxia at ∼2500 m during resting (*P *= 0.013, η^2^_p_ = 0.385), but not during exercise phases (*P *= 0.393, η^2^_p_ = 0.081). As shown in Table 6, neither total transfer entropy during rest nor during exercise significantly predicted the occurrence of symptoms within the same experimental condition.

**TABLE 5:** Network similarities at individual level (computed by transfer entropy values) and symptoms occurrence, of which headache was considered the key one.

Sex	Weighted Jaccard Similarity Index									Symptoms					
	Rest–exercise			CTR–HY1		CTR–HY2		HY1–HY2		CTR		HY1		HY2	
	CTR	HY1	HY2	Rest	Ex.	Rest	Ex.	Rest	Ex.	S	H	S	H	S	H
♀	0.388	0.270	0.330	0.306	0.135	0.376	0.327	0.295	0.144	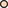	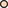	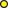	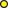	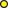	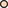
♀	0.499	0.510	0.501	0.589	0.419	0.586	0.508	0.512	0.386	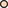	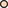	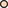	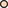	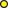	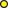
♀	0.535	0.330	0.377	0.431	0.513	0.397	0.466	0.478	0.469	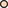	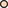	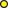	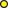	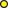	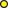
♀	0.462	0.125	0.342	0.187	0.442	0.357	0.474	0.134	0.643	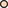	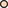	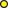	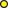	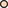	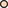
♀	0.326	0.042	0.445	0.032	0.482	0.137	0.459	0.079	0.379	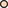	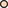	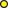	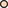	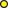	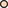
♀	0.200	0.098	0.285	0.256	0.395	0.288	0.216	0.328	0.146	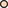	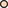	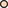	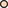	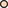	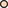
♂	0.268	0.116	0.262	0.111	0.035	0.231	0.270	0.076	0.024	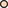	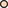	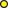	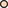	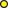	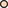
♂	0.367	0.165	0.537	0.200	0.326	0.392	0.317	0.223	0.216	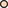	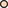	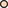	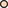	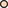	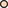
♂	0.533	0.296	0.317	0.297	0.422	0.482	0.382	0.325	0.358	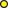	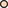	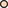	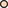	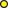	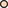
♂	0.097	0.280	0.199	0.250	0.408	0.286	0.402	0.204	0.535	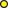	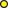	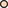	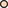	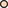	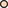
♂	0.369	0.317	0.307	0.362	0.461	0.418	0.478	0.391	0.315	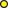	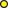	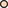	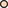	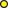	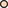
♂	0.250	0.274	0.407	0.330	0.227	0.337	0.298	0.427	0.386	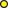	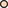	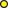	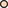	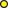	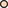
Group	0.358	0.235	0.359	0.279	0.355	0.357	0.383	0.289	0.333	4	2	6	3	8	2

Peach‐pink dots mean negligible symptom occurrence while yellow dots mean light symptom occurrence. CTR, control condition; HY1, hypoxia at simulated altitude of ∼2500 m; HY2, hypoxia at simulated altitude of ∼3,500 m; Ex, exercise; S, symptoms; H, headache.

**TABLE 6:** Binomial regressions of transfer entropy at rest and exercise to the occurrence of symptoms within the same experimental condition.

	VIF	*P*	AUC	*R* ^2^_McF_	AIC	BIC		*P*	Odds ratio
Control	1.272	0.795	0.594	0.030	20.82	22.27	Rest	0.815	0.959
							Exercise	0.512	1.186
Hipoxia1	1.066	0.365	0.694	0.121	20.62	22.08	Rest	0.232	0.803
							Exercise	0.911	0.982
Hypoxia2	1.107	0.550	0.625	0.078	20.08	21.53	Rest	0.369	1.163
							Exercise	0.415	0.848

AIC, Akaike information criterion; AUC, area under curve; BIC, Bayesian information criterion; Hypoxia1 and 2, simulated altitude of ∼2500 and ∼3500 m; VIF, variance inflation factor; *R* ^2^_McF_, McFadden's *R* ^2^.

The binomial regression of SF ratio to the occurrence of symptoms within the same experimental condition revealed that neither during control (*P *= 0.285, AUC = 0.641, McFadden's *R* ^2^ (*R* ^2^_McF_) = 0.075, AIC = 18.13, BIC = 19.10, odds ratio = 0.898) nor in H1 (*P *= 0.528, AUC = 0.583, *R* ^2^_McF_ = 0.024, AIC = 20.24, BIC = 21.21, odds ratio = 1.034) nor in H2 (*P *= 0.175, AUC = 0.750, *R* ^2^_McF_ = 0.120, AIC = 17.44, BIC = 18.41, odds ratio = 1.083) was the desaturation normalized on $F_{iO_{2}}$ able to predict the occurrence of symptoms.

## 4 DISCUSSION

The organ‐level response of humans exposed to hypoxia has been an intriguing field of research for the past century. This study aimed to provide novel insights by complementing classical analytical approaches with network models and to fill some gaps of simulated procedures in hypoxia. Individual responses to hypoxia varied across participants, although some physiological similarities exist for $P_{\mathrm{ET}O_{2}}$, which decreased as expected as a function of hypoxia and, to a lesser extent, ${\dot{V}}_{O_{2}}$, which partially increased during hypoxia. ${\dot{V}}_{O_{2}}$ also exhibited a greater increase during isometric exercise over resting in hypoxic conditions. This result was not observed for HR and ${\dot{V}}_{E}$, which increased similarly from resting to isometric exercise in the three conditions. Considering the trend of RF and *V*_T_, which increased similarly during exercise and whose increment was not affected by hypoxia, we can conclude that our participants showed an appropriate response, since the impaired response would have featured a more pronounced increase in RF rather than *V*_T_ (Bondi & Verratti, 2023).

Network properties were more similar comparing Control and Hypoxia at 3500 m to each other than to Hypoxia at 2500 m; therefore, the low‐grade hypoxia unexpectedly promoted changes in physiological interconnectedness more than mild grade hypoxia. When comparing network diagrams for different experimental conditions, there was a greater similarity between Control and Hypoxia at 3500 m rather than between low grade hypoxia at 2500 m and other experimental conditions. Low grade hypoxia shows decreased interconnectivity within the network during both rest and exercise conditions; this suggests that information converges to a hidden node (a node which is not included in this network map of the cardiorespiratory control system). Previous reports suggest that this node could be peripheral oxygen saturation ($S_{pO_{2}}$); a study by Jiang et al. has shown that $S_{pO_{2}}$ fluctuations during normobaric hypoxia carry information about integrated cardiorespiratory control, and network mapping has shown hypoxia to be the hub of the cardiorespiratory network with significant bidirectional information transfer with all nodes (RF, HR, *V*_T_, ${\dot{V}}_{E}$, $S_{pO_{2}}$, $P_{\mathrm{ETC}O_{2}}$, $P_{\mathrm{ET}O_{2}}$) within the network (Jiang et al., 2021). Furthermore, another recent study which assessed network mapping during hypoxia and exercise, alone and combined, has found $S_{pO_{2}}$ to be the main disseminator of information during exercise and the network hub (main receiver and disseminator of information) during hypoxic conditions (Morandotti, Rigny et al., 2026). However, inaccuracy of $S_{pO_{2}}$ measurements during low perfusion, body movement and low oxyhaemoglobin saturation (Prosperi et al., 2023) currently limit the use of $S_{pO_{2}}$ for hypoxia‐induced analyses.

When comparing the HY1–exercise and HY2–exercise network diagrams and indices, it can be observed that HR has the highest OD value of all nodes, meaning that HR is a prominent disseminator of information during hypoxia and exercise combined; the state of other physiological variables in the cardiorespiratory control network is impacted by the state of HR. HR's high OD value could represent the coordination of responses to optimize oxygen delivery upon changes in cardiac output.

A similar pattern can be observed for RF, which has a high OD value in some experimental conditions involving hypoxia and exercise. This could also imply an important role of changes in respiratory rate in coordinating a response to optimize oxygen delivery throughout the body.

A similarity between control–exercise and HY2–exercise can also be observed; in both experimental conditions the node which disseminated the most information is $P_{\mathrm{ET}O_{2}}$, and the node which received most information is RF, with information transfer occurring bidirectionally between both nodes, and higher TE values between RF and $P_{\mathrm{ET}O_{2}}$ (increased transfer of information) during Control–Exercise and HY2–Exercise compared to their respective rest conditions. At least for HY2, this may represent the exercise‐induced potentiation of the hypoxic ventilatory response (HVR), which consists of the cardiorespiratory system's increased respiratory frequency upon detection of low oxygen levels during hypoxia being further increased by exercise (Oliveira et al., 2024).

It is worth mentioning the heterogeneity of responses in spite of a homogeneous group of participants (at least in terms of age, health and previous exposures to hypoxia). The heterogeneity in acute response to normobaric hypoxia and isometric exercise align with other observations, such as those on hypoxia and bed‐rest in response of a short‐term (10 and 21 days) exposure (Royal et al., 2021; Salvadego et al., 2021). Among putative factors, inter‐individual differences in chemosensitivity to hypoxia, and genotypic and phenotypic differences in stress–response pathways may be evoked. These arguments fall within the wider interest on characterizing the stress response across levels of biological organization (e.g. Heidinger & Wada, 2019).

In empirical observations related to environmental physiology, we noticed a very diverse response on $S_{pO_{2}}$ time series during hypoxia exposure across participants. The fluctuations that define the $S_{pO_{2}}$ variability can represent a proxy for coupled systems of cardiorespiratory control (Bhogal & Mani, 2017). However, technical limitations leading to inaccuracy of measurements during low perfusion or body movement, along with the contextual biases due to low performances of pulse oximeters in case of low $S_{pO_{2}}$ (Prosperi et al., 2023), still hamper the use of $S_{pO_{2}}$ variability as a proxy of individual heterogeneity in physiological response to hypoxia.

Although continuous measurement of $S_{pO_{2}}$ was not the purpose of this study, we measured spot $S_{pO_{2}}$ to be consistent with hypoxic stress studies. In particular, $S_{pO_{2}}$ to $F_{iO_{2}}$ ratio (SF) can be used rather than fixed approaches based on $F_{iO_{2}}$ alone because this index includes the bodily response ($S_{pO_{2}}$) normalized to the external stimulus ($F_{iO_{2}}$). In our participants, as expected SF increased as a function of hypoxia. Our results of SF ratio can be superimposed to those of Soo et al. (2020) on hypothetical $S_{pO_{2}}$ response to a hypoxia test at $F_{iO_{2}}$ of 0.13, 0.15 and 0.17 related to their virtual participant with the least desaturation. SF was developed to monitor the response of ventilated patients. To our knowledge, whether or not SF ratio, as the individual response to lower available oxygen, is associated to the occurrence of symptoms in normobaric hypoxia is still unknown. In our participants, SF was not associated with the occurrence of symptoms. Neither was TE. One question that arises is: are placebo/nocebo effects more prominent in hypoxic conditions? Further studies may focus on severe symptoms, severe hypoxic conditions and prolonged exposure.

### 4.1 Limitations

The networks were plotted based on the available data, which means there may be hidden nodes which were not visible in this study's network diagrams and could play a role; this could result in potentially significant information transfer links being concealed. For example, $S_{pO_{2}}$ time series were not recorded in this study, which may explain why the physiological network in HYP1 appears to have less information transfer between the nodes. Future studies should evaluate which nodes are essential in physiological network mapping during hypoxia and exercise challenges. While the physiological network mapping used in this study provides valuable insights into integrative physiological control, it also has limitations that warrant further investigation. For instance, transfer entropy is inherently pairwise, which can lead to two issues in multivariable settings: (1) overestimation due to redundancy, where the same information shared across multiple sources is counted more than once, and (2) underestimation due to synergy, where no single source provides complete information, but a combination of sources does (Faes et al., 2015; Morandotti, Rigny et al., 2026).

The sample size of this study represented a limitation to connect symptoms with physiological network measures, particularly considering the heterogeneity of the responses. The results of this study can be used for statistical power calculation prior to larger study to fully investigate the relationship between symptoms and integrated physiological control.

### 4.2 Troubleshooting and perspectives

The data we obtained preliminarily on CO_2_ dynamics during different levels of oxygen, as well as those obtained by modelling temperature and relative humidity, which both served as a quality control for the inter‐condition comparisons, can also serve as reference basis for further studies using hypoxic tents. Specific $S_{pO_{2}}$ to $F_{iO_{2}}$ (SF) ratio profiles can provide primary references for optimizing hypoxic stress tests and hypoxic training sessions. Continuous data acquired during combined exposure to hypoxia and exercise can expand current databases, as done for Sport DB 2.0 (Romagnoli et al., 2023), thus providing signals for developments and validation of network analysis methods. This gap is evident in the most famous database for complex physiological signals, that is, Physionet (Goldberger et al., 2000); no data are present for either normobaric or hypobaric conditions. The field of network physiology, which aims to establish the human physiolome as a large‐scale reference from multiple systems for mapping physiological systems interactions in different states, conditions and diseases (Ivanov, 2021), cannot leave this gap.

The procedures proved to be safe. Troubleshooting included climatic management inside the tent (increased temperature and decreased relative humidity) and possible issues with data acquisition from the devices due to altered air composition. Accurate measurement of $F_{iO_{2}}$ and $F_{\mathrm{iC}O_{2}}$ can be implemented in bioenergetic studies during simulated hypoxia to correct data from metabolimeters calibrated outside the tent. The time series of ${\dot{V}}_{O_{2}}$ exhibited more smoothness than $P_{\mathrm{ET}O_{2}}$ and $P_{\mathrm{ETC}O_{2}}$ and did not necessitate impactful data treatment, being thereby a valid and easily manageable variable to be included in hypoxic simulation studies. However, great caution must be used in the interpretation of metabolimeter data, and great attention must be paid to instrument calibration, data observation and offline processing. Our network approach, given the recommendations of this troubleshooting report, can be used for novel and physiologically relevant insights in hypoxic exercise testing.

### 4.3 Conclusions

Heterogeneity of physiological responses across a homogeneous group of participants (healthy young people not accustomed to hypoxia) represents a useful insight to be considered for its potential impact on sports science and environmental physiology, thus extending the need of pursuing individual‐based approaches to capture physiological responses to stressful stimuli. Compared to mild‐grade hypoxia, low‐grade hypoxia induced more changes in physiological connectivity. Rest–exercise networks were less similar to each other during a simulated altitude of 2500 m than of normoxia or a simulated altitude of 3500 m. Neither TE during rest nor during exercise, nor the $S_{pO_{2}}$/$F_{iO_{2}}$ ratio predicted the occurrence of hypoxia‐induced symptoms. Understanding the interconnectedness and adaptiveness of physiological systems and subsystems through the lenses of complex systems science, graph theory and dynamic systems theory raises the possibility of unveiling novel insights for translational sports medicine and applied physiology. In the future, network mapping and network indices could be used to define a healthy or normative response to specific stressors and then detect outlier responses through network indices which could be associated with states of disease by comparing an individual patient's/athlete's response to the defined normative response. Innovative approaches for understanding emergent behaviours, state transitions and physiopathological trajectories are leading to meaningful implications for sport, fitness and lifestyle.

## AUTHOR CONTRIBUTIONS

Conceptualization, Danilo Bondi and Cecilia Morandotti; Methodology, Danilo Bondi, Cecilia Morandotti and Vittore Verratti; Formal Analysis: Danilo Bondi and Cecilia Morandotti; Investigation, Danilo Bondi, Salvatore Annarumma, and C.S.; Writing—Original Draft, Danilo Bondi and Cecilia Morandotti; Writing—Review & Editing, Salvatore Annarumma, Carmen Santangelo, Tiziana Pietrangelo, Stefania Fulle and Vittore Verratti; Funding Acquisition, Vittore Verratti; Resources, Tiziana Pietrangelo, Stefania Fulle and Vittore Verratti; Visualization: Danilo Bondi and Cecilia Morandotti; Supervision, Vittore Verratti. All authors have read and approved the final version of this manuscript and agree to be accountable for all aspects of the work in ensuring that questions related to the accuracy or integrity of any part of the work are appropriately investigated and resolved. All persons designated as authors qualify for authorship, and all those who qualify for authorship are listed.

## CONFLICT OF INTEREST

The authors have nothing to report.

## FUNDING INFORMATION

No funding has been received for this work.

## Supporting information



Figure S1. Network diagrams where node size is directly proportional to OD values, and edge thickness is directly proportional to TE values.

## Data Availability

Data will be available upon reasonable request
