# Supplementary material for: How do physiological networks respond to normobaric hypoxia and isometric exercise?
Source: Exp Physiol. 2025 Nov 20;111(4):1904–18. doi: 10.1113/EP093077 (PMC13140655; doi:10.1113/EP093077)
Supplement: Supplementary file 1 — Figure S1. Network diagrams where node size is directly proportional to OD values, and edge thickness is directly proportional to TE values. [file EPH-111-1904-s001.docx]

**How do the physiological networks respond to normobaric hypoxia and isometric exercise?**

*Danilo Bondi^#^, Cecilia Morandotti^#^, Salvatore Annarumma, Carmen Santangelo, Tiziana Pietrangelo, Stefania Fulle, Vittore Verratti*

**Appendix**


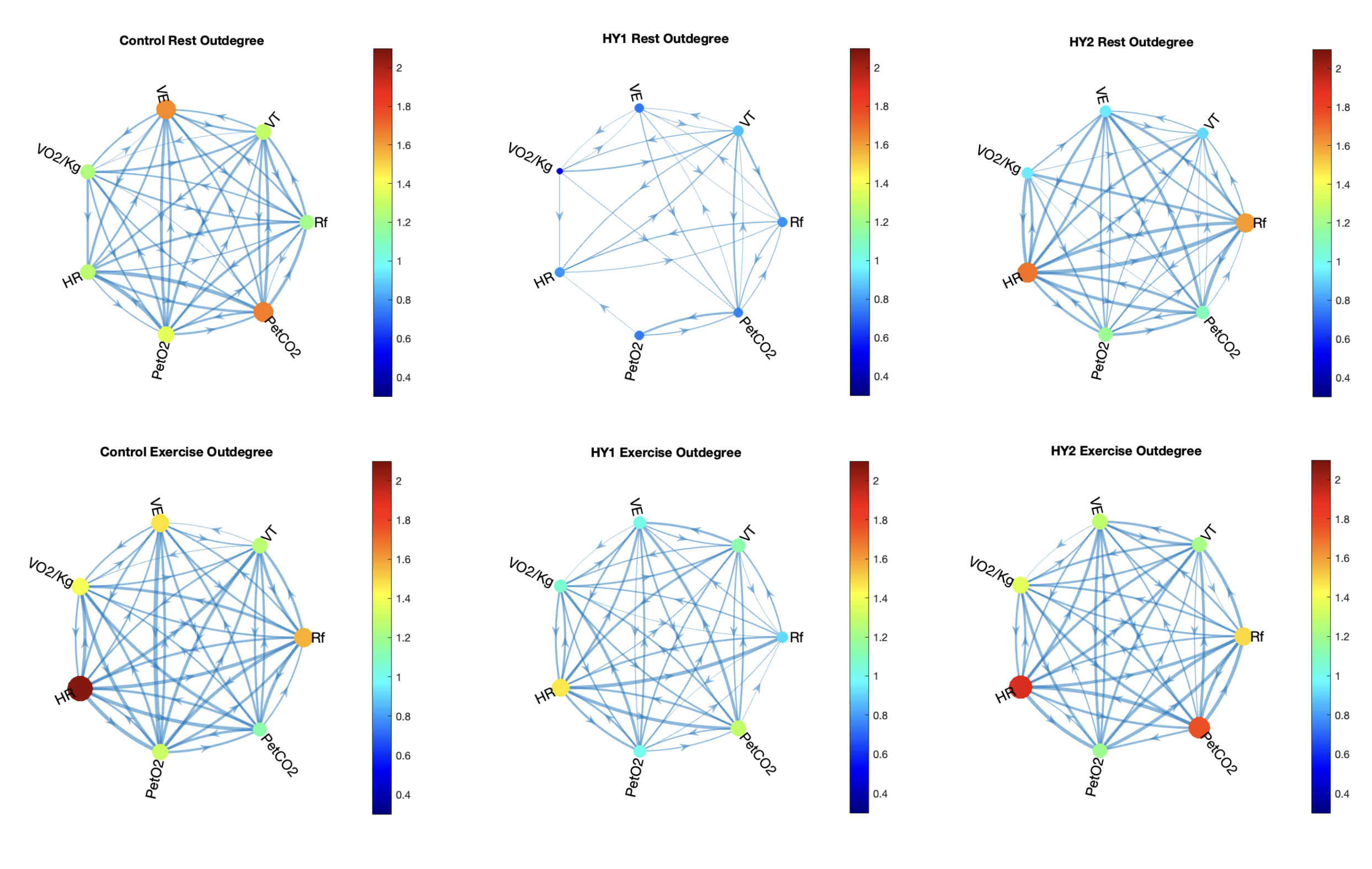


**Figure A1**: Network Diagrams where node size is directly proportional to OD values, and edges thickness is directly proportional to TE values.
